# Editorial: Machine learning and psychosis: Diagnosis, prognosis and treatment

**DOI:** 10.3389/fpsyt.2023.1133072

**Published:** 2023-02-21

**Authors:** Enrico D'Ambrosio, Anees Abrol, Alessandro Pigoni

**Affiliations:** ^1^Department of Translational Biomedicine and Neuroscience (DiBraiN), University of Bari Aldo Moro, Bari, Italy; ^2^Department of Psychosis Studies, Institute of Psychiatry, Psychology and Neuroscience, King's College London, London, United Kingdom; ^3^Tri-institutional Center for Translational Research in Neuroimaging and Data Science (TReNDS), Georgia State University, Georgia Institute of Technology, and Emory University, Atlanta, GA, United States; ^4^Department of Neurosciences and Mental Health, Fondazione IRCCS Ca' Granda, Ospedale Maggiore Policlinico, Milan, Italy; ^5^Social and Affective Neuroscience Group, MoMiLab, IMT School for Advanced Studies Lucca, Lucca, Italy

**Keywords:** machine learning (ML), psychosis, schizophrenia, electronic health records, genetics

Psychosis is a severe condition associated with considerable disability that affects patients and their families, causing substantial economic burdens in terms of lost productivity and direct costs (medications, hospitalizations, etc.) (1, 2). Individuals affected by psychosis still suffer from a low recovery rate and have a high likelihood of relapse. Moreover, patients affected by psychosis suffer from premature mortality due to cardio-metabolic complications and a higher risk for suicide (3–5).

For these reasons, psychosis prevention and early treatment are public health priorities. However, to date, psychosis still presents many unresolved challenges in diagnosis, identification of specific biomarkers, and treatments, especially in its early stages. Diagnoses are often blurred at presentation, and subgroups of individuals do not achieve a symptomatic and functional recovery, despite receiving specialized care. An increasing amount of evidence supports implementing early detection and intervention in psychotic disorders (6), with benefits in terms of a lower rate of relapse and overall better quality of life and functioning (7).

A precise definition of outcomes and response to treatments assessed readily on presentation would certainly impact patient management. However, so far, studies have struggled to identify valid biomarkers of psychosis trajectories and treatment response (8). Within the many strategies to improve the detection and outcomes of psychosis, machine learning (ML) techniques have seen tremendous development as they are resourceful in handling complex problems. ML are data-driven methods that deal with multivariate patterns by going beyond group-level analyses (9). These techniques have the potential to learn subtle data attributes, analyze massive data sets and combine different modalities (e.g., neuroimaging, clinical data, genetics, etc.) with the final aim of informing clinical decisions associated with diagnosis, prognosis, and treatment in psychiatry. ML can be employed to uncover patterns of risk and biomarkers, helping in early detection, understanding neurobiological heterogeneity, defining subgroups and endophenotypes, and predicting disease trajectory (10, 11).

Therefore, this Research Topic examines how modern computational techniques could contribute to clinical practice in addressing the unmet needs of patients with psychosis in terms of differential diagnoses, treatment, and outcome prediction. The qualified papers assessed the topic from different points of view, from genes to clinical and imaging data, proving how multifaced and adaptable ML techniques are to multiple contexts.

One of the significant advantages of ML tools is the possibility of handling huge amounts of data, such as those obtained from electronic health records (EHRs). EHRs represent a great source of information but often present a lack of standardized instruments, poor quality of the data, and a volume of information difficult to analyze. In their novel paper, Lee et al. assessed the possibility of using EHRs data to predict a 1-year relapse of psychosis. Moreover, they enriched their prediction, including natural language processing (NLP) data from clinical notes and psychological tests. NLP is another exponentially growing field within the bigger ML domain, with the possibility to handle data from clinical notes and patients' records (12). Finally, Lee et al. tested their prediction on an out-of-distribution sample from another hospital. In ML model creation, it is paramount to bear in mind the risk of overfitting the data, creating a model that works very well in the tested population but poorly generalizes to other samples. In the quest to create a tool to implement in clinical practice, overfitting should be avoided. One of the most valuable ways to test the generalizability of a model is by using a completely distinct sample with different characteristics (9), as done by Lee et al..

As our knowledge of the genetic base of psychiatry disorders grows (13), it also increases the need to put these genes in the proper context, clustering them in biological pathways that can help us better understand the pathophysiology of psychosis. From this perspective, Liu et al. performed ML analyses to assess differential gene expression in individuals with schizophrenia and healthy controls, using RNA-seq data from the dorsolateral prefrontal cortex and amygdala. This approach led to an expansion of established schizophrenia-associated gene networks involved in the neurodevelopmental processes implicated in the neurobiology of schizophrenia, including glutamatergic, serotonergic, and dopaminergic synapse pathways, neuroactive ligand-receptor interaction networks, and cAMP signaling pathways.

As for genetics, imaging studies have taken great advantage from ML and strategies for automatic diagnostics in recent years (14). Not only classification (15) but also subtyping of psychotic disorders and prognostic studies (10) have been performed. Haas et al. used structural neuroimaging data to compute the brain age of patients with early-stage schizophrenia and healthy individuals and then clustered patients using multidomain cognitive data. In this way, they distinguished a cluster of cognitively impaired patients from cognitively spared individuals. Interestingly, both local and global brain age were significantly higher in the cognitively impaired population, suggesting that subjects with more severe symptoms also present a steeper decline through the years.

Finally, we are pleased to include articles discussing the challenges and pitfalls of these techniques, such as the perspective article by Smucny et al., who review methods to overcome problems with deep learning tools. Typically, deep learning methods need large datasets and are considered “black boxes,” obscuring their use in medical imaging applications. The authors describe three novel approaches that may help accelerate the incorporation of deep learning into the clinic. They first introduce two methods that can reduce the amount of training data required to develop accurate models: transfer learning and data augmentation. Next, they assess the complex topic of explainable artificial intelligence, tools that reveal what features (and in what combinations) deep learners use to make decisions. Such techniques might help resolve the “black box” problem, a critical step to validating ML tools in clinical practice.

Taken together, the papers included in this Research Topic illustrate how machine learning techniques have the potential not only to improve our understanding of the neurobiology of psychosis but also to contribute to crucial aspects of the clinical practice, such as diagnosis, prognosis, and treatment of this disorder.

## Author contributions

All authors listed have made a substantial, direct, and intellectual contribution to the work and approved it for publication.
